# Evaluation of the Acceptability and Feasibility of Stress Mitigation Education and Support Delivered via Telehealth for People After Road Traffic Musculoskeletal/Orthopedic Injury

**DOI:** 10.1007/s10926-024-10258-z

**Published:** 2024-11-29

**Authors:** Alison Sim, Amy G. McNeilage, Trudy Rebbeck, Michele Sterling, Michael Nicholas, Sarah Donovan, Melita J. Giummarra, Claire E. Ashton-James

**Affiliations:** 1 Sydney School of Health Sciences, Faculty of Medicine and Health, The University of Sydney, Sydney, Australia; 2 Northern Sydney Local Health District, Sydney, Australia; 3 RECOVER Injury Research Centre, Faculty of Health and Behavioural Sciences, The University of Queensland, Brisbane, Australia; 4 Department of Neuroscience, School of Translational Medicine, Monash University, Melbourne, Australia; 5 Caulfield Pain Management and Research Centre, Caulfield Hospital, Caulfield, Australia; 6 Sydney Medical School (Northern Clinical School), Kolling Institute, The University of Sydney, Lvl 2 Douglas Building, St Leonards, 2065 Australia

**Keywords:** Motor vehicle accident, Road traffic Injury, Intervention, Distress, Telehealth, Digital health

## Abstract

**Purpose:**

To evaluate the acceptability and feasibility of a consumer co-designed telehealth intervention which aimed to reduce claimant distress by providing pain management strategies, informational and social support to people who had made a compensation claim following road traffic musculoskeletal injury.

**Methods:**

Eleven claimant participants who were at risk of a poor outcome completed the intervention in a one-on-one setting with the same clinician delivering the program across all sessions.They were interviewed about their experience (acceptability and feasibility including the use of telehealth). Clinicians who delivered the intervention also completed an anonymous feedback survey exploring their experiences delivering the intervention. Interviews were transcribed verbatim and thematic analysis was conducted.

**Results:**

There were four themes which broadly related to the acceptability and feasibility of the intervention as well as the perceived benefits: (1) knowledge is power, (2) healing with social connection, (3) further along than I would have been, and (4) telehealth was acceptable and feasible.

**Conclusion:**

The delivery of a co-designed telehealth-delivered stress mitigation intervention to support people with a road traffic musculoskeletal injury was feasible to deliver and acceptable to people who were at risk of a poor outcome. Further research to evaluate the efficacy of the intervention on outcomes such as pain, self-efficacy, and claims costs are needed.

**Supplementary Information:**

The online version contains supplementary material available at 10.1007/s10926-024-10258-z.

## Introduction

People who have experienced road traffic musculoskeletal injury report many issues in the months following injury including disability, difficulty dealing with compensation insurance bodies, trouble accessing rehabilitation, and difficulty dealing with pain [1–3]. While distress and pain following road traffic injury is common [2, 4–8], higher levels of distress following injuries (e.g., anxiety, depression, pain-related distress) are associated with more adverse outcomes, including delayed return to work, higher levels of disability, and an increased risk of developing chronic pain [9–12].

To date, early interventions such as case management strategies [13], stress reduction approaches [14], and psychological therapies [15–17] have improved outcomes such as total claims costs, disability, and return to work experiences. However, other early interventions have been less effective [18–20] suggesting that elements such as the volume of care, mode of delivery, clinician delivering the intervention, and the type of intervention are important to consider in the design and implementation of interventions. The use of co-design methodology for the development of interventions has been demonstrated to improve stakeholder engagement and improve acceptability. [21–23] With several previous interventions demonstrating issues with efficacy, it could be suggested that co-design methodology is necessary in designing such interventions, to ensure that the needs of the population are being accurately addressed.

Previous research has found that risk assessment tools are able to identify those likely to have a poor outcome following injury [12, 24–27]. Examples of these risk assessment tools that have been used following road traffic accident and workplace injury include WhipPredict [26] and Örebro Musculoskeletal Pain Questionnaire (ÖMPQ) [24]. By identifying who is likely to do poorly following injury, risk assessment screening can help to identify those who may benefit from additional help during the recovery process. This targeted approach to selecting who would benefit from early interventions ensures judicious allocation of resources, as those with low risk are likely to recover without additional help.

Our previous research has identified common sources of distress for people who have experienced an injury that occurs in the setting of an insurance claim, such as workplace injuries and those covered by motor vehicle accident insurance schemes (known as compensable injuries) [28]. Interviews with claimants who have experienced a compensable injury, as well as with clinicians who deliver care to such patients, identified that difficulty navigating the claims process, dealing with pain and disability, and delays to care were common sources of distress. These findings are consistent with other qualitative research exploring people’s experiences with compensable injuries [1, 2, 29, 30]. Research has also identified that difficulty accessing care due to factors such as travel distance and clinician availability can be a barrier to adequate treatment [1, 31–34]. With the Covid-19 pandemic prompting the use of telehealth services out of necessity, there has been a growing acceptance of the use of telehealth by both patients and clinicians [35, 36]. Further, delivering healthcare via telehealth can help to address the inequalities in access to healthcare experienced by rural people, compared with urban dwellers [37–39]. Finally, there is emerging evidence to suggest that telehealth-delivered interventions can be helpful in improving mental health outcomes [40–42].

The primary objectives of this study were to assess the acceptability and feasibility of a telehealth-delivered intervention to provide additional support for people who have experienced a recent road traffic injury in Victoria, Australia.

## Materials and Methods

The study was approved by The University of Sydney Human Research Ethics Committee [Reference: 2022/685].

### Study Design and Setting

This was an acceptability and feasibility study of an early intervention to mitigate distress early following motor vehicle injury.

### Participants and Recruitment

Eligible claimant participants were people 18 years or older who had experienced a musculoskeletal or orthopedic injury following road traffic injury on a Victorian road within 16 weeks. Additionally potential participants were required to have lodged a claim with the Transport Accident Commission (TAC), Victoria’s no-fault road traffic accident insurance scheme. Recruitment of claimants was done via waiting room advertisement in primary care clinics (general practitioner, physiotherapy, osteopathy) in Victoria, Australia, as well as social media advertising (convenience sampling). The eligibility of prospective participants who volunteered was confirmed by a research team member (AS) via phone or email. Detailed study information was provided, and prospective participants then provided informed consent before completing a risk screening tool, the Örebro Musculoskeletal Pain Screening Questionnaire—Short Form (ÖMPSQ-SF). Prospective participants who scored above 50 out of a total of 100 on the ÖMPSQ-SF, were offered the telehealth intervention. This cut-off score of 50 has previously been identified as providing optimal sensitivity and specificity in predicting delayed return to work following injury [24]. Participants who scored below 50 and were deemed low risk were offered existing digital resources including information on evidence-based recovery strategies [43] as well as direction to the TAC website.

Clinician participants were recruited through the researcher’s network and were eligible if they were registered with the Australian Health Practitioner Regulation Agency (AHPRA), had further training and experience in pain management and regularly managed people with compensable injuries.

#### Claimant Participant Characteristic Data

Claimant participant characteristic data was obtained via survey prior to the first session and included demographic information as well as baseline pain and psychological measures using the Brief Pain Inventory (BPI), [44] Pain Self Efficacy Questionnaire (PSEQ), [45] Pain Catastrophising Scale (PCS), [46] Depression Anxiety and Stress Scale 21 (DASS21), [47] Örebro Musculoskeletal Pain Screening Questionnaire_SF (ÖMPSQ-SF), [24] and Short Form Survey 12 (SF-12) [48]. Claimant demographic information was collected to provide context for their experiences including age and gender, geographical location, employment status, and education level.

### Pilot Intervention

Development of the intervention was undertaken using a co-design process. This process, including the resulting intervention has been published elsewhere [49]. A five-session telehealth-delivered intervention was delivered weekly over a five-week period (Table 1). The sessions were delivered in a one-on-one setting to claimant participants and the same clinician delivered all five sessions to an individual claimant to ensure consistency. Two seven-minute informational videos on the claims process and pain self-management strategies were delivered during the first two sessions. The choice of information to include in the claims process video was based on the co-design process and previous qualitative research undertaken by the research team [28]. This research explored both claimant experiences of the claims process following injury and clinician experiences delivering care to patients in these settings. The video shown in the first session contained information on the claims process including how to apply for treatment and rehabilitation services, tips for navigating the insurance processes, and information to help set expectations (See Table 2 for content topics contained in the first video and Appendix A for the script of the video). Most of the content included in the video was publicly available from the TAC website, however some of the tips provided, such as those around setting expectations for calling the TAC, were included based on the previous qualitative work and the co-design input.

**Table 1:** Outline of intervention

Week/Session	Content	Length of session
1	• Provision of social support through listening and validation • Delivery of claims management informational video (7 min) • Identification of claims management needs based on video information • Claimant goal setting in response to claims information	1 hour
2	• Provision of social support through listening and validation • Review of claimant progress with claims management tasks • Delivery of pain self-management strategies video (7 min) • Identification of opportunities for use of pain self-management strategies	1 hour
3,4,5	• Provision of socio-emotional support through listening and validation • Review of claimant progress with claims management tasks • Review of claimant progress with pain self-management strategies • Review of needs or opportunities related to claim management or pain self-management	30 minutes

**Table 2:** Content topics included in video shown in first session

Content Topic
What is the TAC and how is it funded?
Direction to access information via the TAC website
Information about the myTAC app
Information about approvals processes for treatment, tests, and other medical services
Information about payment requirements for allied health and other healthcare providers including how to find providers
How to access other services that might be required in the setting of an injury or disability
Information on independent medical examinations
Suggestions for contacting the TAC
Links to other support services

A second video with information on pain self-management was produced to be viewed during the second session. The video included evidence-based strategies for non-pharmacological acute pain care such as distraction and relaxation techniques as well as encouraging social connection. The content topics for this video can be seen in Table 3 and the script for this video can be viewed as Appendix B.

**Table 3:** Content topics for video shown in second session

Content Topic
Brief educational information about the acute pain response
Using both pharmacological and non-pharmacological strategies to manage pain
Distraction techniques to reduce pain
Strategies to induce relaxation
Strategies for social connection
Information about the links between mood and pain
Strategies to boost mood
Progressive muscle relaxation
Information about movement and pain

A welcome letter (Appendix C) was provided to participants prior to their first session. It provided information about the intervention and contained links for both videos so that claimant participants could watch the videos at any time to access the information.

The intervention was delivered by experienced allied health clinicians including physiotherapists, osteopaths, and an occupational therapist. These professions were chosen as they all have a role in treating people with compensable injuries. The choice to use a wide variety of professions also helps with the scalability of the intervention. Training for the clinicians was provided in a one-day workshop. Clinicians were instructed during the sessions to allow adequate time to listen to the participant’s story and any issues they were facing following injury and respond to them using validation, thus providing a high level of socio-emotional support. They were then instructed to encourage participants to identify strategies presented in the videos that may help resolve the specific challenges or unmet needs that participants shared regarding their compensation claim, distress, or pain that could be used in the time between sessions. Clinicians would then review the progress made by claimant participants to engage with the identified strategies in subsequent sessions and use motivational interviewing approaches [50] to help the claimant achieve these tasks. This involved clinicians paying attention to the claimant participant’s natural language around desire for behavioral change or activation. Once a goal was collaboratively identified, clinicians used prompting strategies to allow the participants to explore why this change was important to them. They would then collaboratively explore how this might be undertaken [50, 51].

A large emphasis on building self-efficacy through identification of needs and the use of problem-solving strategies building on the informational video content was encouraged by the clinicians in the intervention. Self-efficacy plays an important role in how an individual persists when experiencing difficulty. Clinicians used the theories of self-efficacy to encourage claimant participants to practice and master pain self-management and distress management techniques. By engaging with claims-related tasks that had been identified as being important, participants had opportunities to experience success in these tasks. This sense of success is completing one task helps build self-efficacy, predicting success with engaging with and addressing future tasks [52].

The research team recognized that claimant participants my present with high levels of psychological distress given the nature of experiencing motor vehicle related trauma and injury. To ensure safety of claimant participants and to prevent clinicians from working outside of their scope of practice, claimant participants were required to remain in contact with their existing team of caregivers including their general practitioner (GP). Training procedures for the allied health clinicians detailed referral protocols for claimant participants who were experiencing high levels of distress including suicidal ideation.

### Data Analysis

#### Acceptability and Feasibility Measures

Claimant participants were interviewed by a researcher (CAJ) on Zoom following completion of the intervention. Interviews lasted 20–40 min. The semi-structed interview was guided by a series of open-ended questions that allowed the participant to provide detailed feedback on what they felt was important (see Appendix D for the final interview guide). The interview aimed to understand the participant’s experience of the intervention, the perceived usefulness of the intervention, the likelihood of recommending the intervention to others, and any suggestions for improvement of the intervention. Barriers to engagement, timing of the sessions, issues with scheduling, and any technical issues with accessing the video consultations were also explored. The interviews were recorded and transcribed verbatim. Participants were de-identified by the use of a pseudonym and identifying details were removed. Transcripts were not returned to participants for comment or correction. Coding was conducted using the highlight and comment function in Word. After piloting with three participants we found that participants understood the questions and provided information that elicited responses that addressed the research questions. No changes were made to the interview guide at this point and the data from these pilot interviews was included in the main analysis.

Braun and Clarke’s six-phase framework for thematic analysis was used for analysis of the data [53, 54]. This approach was chosen as it allowed for deep exploration of participant experiences with the intervention. The study was based on a social constructionist epistemology, which assumes that individuals develop knowledge through their interactions with the world, creating meaning from their experiences with others and their environment. Two authors (AS and CAJ) independently reviewed three transcripts and generated codes using an inductive process. Codes were then compared and refined. The remaining four transcripts were then coded by a single researcher (AS). Development of the themes from the codes was then undertaken collaboratively by both researchers (AS and CAJ). To enhance trustworthiness of the findings, multiple strategies were employed. Detailed documentation of the research processes, including of the coding and thematic process helped to ensure methodological transparency. Regular peer meetings aimed to enhance the credibility of the interpretations and reduce potential bias introduced by the two authors interacting with the data.

This study adhered to the Consolidated Criteria for Reporting Qualitative Research (COREQ) to ensure rigorous and transparent reporting of our research processes [55]. The COREQ checklist, which outlines 32 criteria covering research team reflexivity, study design, and data analysis and reporting, served as a guiding framework throughout the study. For added transparency, the completed COREQ checklist is included in Appendix E.

#### Clinician Participant Data

Clinicians were asked to complete an anonymous survey at the end of the data collection period. Five open-ended questions were asked of the clinicians aiming to explore their experiences in delivering the intervention (Appendix F):

- “Tell us about your experience delivering the intervention.”
- “Would you recommend this intervention to patients? Please elaborate.”
- “Would you recommend this intervention to colleagues to deliver as a facilitator? Please elaborate.”
- “What was your experience with participants’ level of engagement with the intervention? Please elaborate on why engagement was high or low.”
- “Did you have any suggestions as to how the intervention could be improved?”

Responses to the these open-ended questions were analyzed using an inductive approach to thematic analysis [54]. The results of the survey questions were read several times and initial codes were noted (AS). A more detailed coding process was undertaken (AS) and reviewed by a second author (CAJ). Subsequently, related codes were grouped into higher-order themes (AS and CAJ). Refinement of the themes was undertaken by the researchers (as and CAJ) using an iterative process. The intention was to compare and contrast the claimant and clinician feedback, hence the interview guide and survey were constructed to be conceptually similar (Appendix D and E). The results of the surveys from the clinicians were triangulated and integrated with the data from the claimant participants to inform the questions around acceptability and feasibility from both points of view.

### Reflexivity Statement

In the process of conducting this qualitative study, it is crucial to acknowledge the subjective lenses through which the data were interpreted [56]. The inter-disciplinary research team consisted of eight members—a female PhD candidate and osteopath (AS), a female social psychologist (CAJ), a female research officer with qualitative expertise (AM), two female clinical physiotherapy researchers (MS and TR), a male pain psychology researcher and clinician (MN), and a female pain specialist (SD)—who all contributed to the design of the intervention and evaluation. The analysis of the data was undertaken by two researchers (AS and CAJ), each bringing unique perspectives and experiences to the interpretation of the findings. The first (AS), an osteopath specializing in treating chronic pain with experience working with patients in compensable settings, approached the research with a desire to address the challenges faced by some their patients. Her clinical experience undoubtedly influenced her interpretation of the data, as her encounters with patients struggling within the compensable care landscape provided prior context. Her perspective may have predisposed her toward identifying particular issues reflective of her own clinical practice. The second (CAJ), with a background as a social psychologist and experience in researching clinicians and claimants in multiple settings, approached the analysis with a broader understanding of the systemic dynamics at play within compensable care settings. Having engaged in prior research with clinicians operating within this domain, she brought insights into the intersecting challenges faced by clinicians, claimants, and insurers. Her perspectives may have fostered a more nuanced appreciation of the structural factors shaping the experiences of compensable care patients. By acknowledging these potential biases and engaging in ongoing reflexivity, we were mindful to enhance the rigor and transparency of our study, ultimately contributing to a more nuanced understanding of the complexities inherent in compensable care contexts.

## Results

Twenty-three people responded to the advertisement and were screened for eligibility. Eleven of these respondents either had scores below 50 on the ÖMPSQ-SF screening questionnaire, declined to participate, or were otherwise not eligible. A total of 11 claimant participants completed the intervention. One participant dropped out after two sessions due to their low time availability. Ten participants were from Melbourne metropolitan regions, and one lived in a rural area. All participants spoke English. No participants attended a session with a support person. All sessions were able to be delivered to participants, although five sessions were delivered over telephone with no video conferencing due to claimant preference or internet connectivity issues. Where this occurred in the first two sessions when videos would normally have been shown, participants either watched the video on a separate device or had watched the video prior to the session. Baseline claimant participant characteristics are reported in Table 4 together with details about individual participant recruitment and other demographic characteristics.

**Table 4:** Claimant participant demographic and recruitment data

No. Pseudonym used	Characteristics	How they learned about the study	Current healthcare providers	Baseline PROMS	
Kate	Female, 40, Metropolitan location, Degree level education, Employed, 9 weeks post-injury	Waiting room sign at local osteopath	Physiotherapist, GP, osteopath	BPI-Pain BPI-Int ÖMPSQ-SF PSEQ PCS DASS SF12-PCS12 SF12-MCS12	5/10 5.5/10 52/100 21/60 35/52 36/42 24 50
Emma	Female, 42, Metropolitan location, Diploma level education, Employed, 10 weeks post-injury	Waiting room sign at local physiotherapy clinic	Physiotherapist, GP, hand therapist	BPI-Pain BPI-Int ÖMPSQ-SF PSEQ PCS DASS SF12-PCS12 SF12-MCS12	7.5/10 8/10 69/100 19/60 24/52 35/42 36 27
Alex	Non-Binary, 31, Metropolitan location, Degree level education, Employed, sick leave, 3 weeks post-injury	Waiting room sign at outpatient department	Outpatient allied health	BPI-Pain BPI-Int ÖMPSQ-SF PSEQ PCS DASS SF12-PCS12 SF12-MCS12	4/10 8/10 63/100 18/60 17/52 38/42 29 33
Charles	Male, 35, Metropolitan location, Degree level education, Employed, sick leave, 2 weeks post-injury	Waiting room advertisement at Priority Primary Care Clinic	GP	BPI-Pain BPI-Int ÖMPSQ-SF PSEQ PCS DASS SF12-PCS12 SF12-MCS12	5 3.5 53/100 35/60 5/52 26/44 38 53
Nicole	Female, 57, Metropolitan location, Unemployed, Education not stated, 4 weeks post-injury	Waiting room osteopathic clinic	Osteopath, GP	BPI Pain BPI-Int ÖMPSQ-SF PSEQ PCS DASS SF12-PCS12 SF12-MCS12	8/10 8/10 86/100 14/60 29/52 39/44 22 32
Andrea	Female, 27, Metropolitan location, Employed, Diploma level education, 10 weeks post-injury	Waiting room osteopathic clinic	Osteopath, GP, myotherapist, acupuncturist, psychologist, physiotherapist	BPI Pain BPI-Int ÖMPSQ-SF PSEQ PCS DASS SF12-PCS12 SF12-MCS12	6/10 7/10 78/100 23/60 11/53 27/44 25 33
Alistair	Male, 42, Metropolitan location, Degree level education, Employed, 13 weeks post-injury	Advertisement in TAC quarterly newsletter	Physiotherapist, chiropractor	BPI-Pain BPI-Int ÖMPSQ-SF PSEQ PCS DASS SF12-PCS12 SF12-MCS12	6/10 6/10 57/100 24/60 26/52 20/44 36 36
Grace	Female, 30, Metropolitan location, Degree level education, Employed, Sick leave, 2 weeks post-injury	Waiting room sign at outpatient department	Physiotherapist, Occupational therapist, GP	BPI-Pain BPI-Int ÖMPSQ-SF PSEQ PCS DASS SF12-PCS12 SF12-MCS12	3.5/10 8/10 57/100 34/60 10/53 23/44 25 46
Bob	Male, 54, Metropolitan location, Year 10 level education, Unemployed, 12 weeks post-injury	Waiting room sign at outpatient department	GP, outpatients surgical, allied health	BPI-Pain BPI-Int ÖMPSQ-SF PSEQ PCS DASS SF12-PCS12 SF12-MCS12	2/10 6/10 66 23/60 24/52 25/44 33 39
Elizabeth	Female, 31, Metropolitan location, Degree level education, Employed, 15 weeks post-injury	Waiting room sign at GP clinic	Physiotherapist, GP, remedial massage	BPI-Pain BPI-Int ÖMPSQ-SF PSEQ PCS DASS SF12-PCS12 SF12-MCS12	3.5/10 6.5/10 55/100 30/60 14/53 16/44 33 54
Simon	Male, 41, regional location, Year 9 level of high school, Unemployed, 14 weeks post-injury	Waiting room sign at outpatient department	Physiotherapy, specialist outpatients, GP	BPI-Pain BPI-Int ÖMPSQ-SF PSEQ PCS DASS SF12-PCS12 SF12-MCS12	6/10 9/10 90/100 23/60 24/52 39/44 22 27

BPI-Pain Brief Pain Inventory pain subscore, BPI-Int brief pain inventory pain interference subscore, PSEQ Pain Self-Efficacy Questionnaire, DASS21 Depression Anxiety Stress Scale 21, SF-12 Short Form Survey 12, MCS-12 Mental Component Score 12, PCS-12 Physical Component Score 12, ÖMPSQ-SF Örebro musculoskeletal pain questionnaire

Four clinicians were recruited (in addition to author AS who delivered the intervention to two participants) and attended the training day. We received four responses to the anonymous feedback survey sent to clinicians following completion of the pilot intervention.

### Clinician Facilitator Demographic Information

Two osteopaths, two physiotherapists, and one occupational therapist delivered the intervention to one or more participant each. All clinicians had further education and experience in the area of pain management. Clinicians had 11 to 33 years experience.

### Participant Experiences of Delivering or Receiving the Intervention

Four themes broadly related to the acceptability, feasibility, and perceived benefits of the intervention, from the perspectives of both the claimants and clinicians: (1) knowledge is power; (2) healing with social connection; (3) further along than I would have been; and (4) telehealth was acceptable and feasible.

#### Knowledge is Power

In learning about the nature of the claims process and the tasks required of them, many participants reported the information gained from the videos was instrumental to their understanding of their role and the requirements to navigate the claims process. Additionally, claimant participants reported that the strategies described in the pain self-management videos provided new options or reminders to engage in strategies to help deal with pain. The knowledge or reminders that were gained from the videos gave participants opportunities to engage in their own recovery process with greater confidence, suggesting that they had increased self-efficacy.

The content of the two informational videos was described by claimant participants to be helpful for several reasons. For some, it provided a reminder or prompt to action claims processes: *“It was quite informative… I thought it was actually quite helpful to show me, remind me things about the TAC”* (Kate). For many others, the information provided in the video was new and was instrumental in helping them to navigate claims process, set expectations, or understand how the TAC could assist their recovery. For example, Grace described that as she was from another Australian state, she had little prior knowledge of the TAC and its role: *“I’d never even heard of the TAC before and obviously in the hospital I heard about it, but I just didn’t know anything… The videos would explain the process.”* For Kate it provided a prompt to action certain administrative tasks before an important cut-off date within the standard claims process: *“There were a few things I didn’t realise. I had to reach out to them… It was great that… I got those prompts and there was a bit of content that was in the videos… that I wasn’t aware of.”* For Simon, the TAC claims information in the video helped set expectations: *“I found [the video] really helpful. It’s helped me a lot, just in terms of managing the TAC and myself and knowing what is expected.”*

For several participants, the claims informational video prompted them to act on an administrative task. It was recognized by the claimants that this prompt and the subsequent action taken had prevented a delay to treatment that was likely inevitable without the knowledge gained from the video information. Kate reflected:

“Being on the front foot, contacting the providers and getting them to instigate the approval process was definitely very helpful for me… Having that extra knowledge of being proactive and reaching out to them instead of waiting for them to reach out to me.”

Emma explained that the sense of being overwhelmed she was experiencing following her accident meant she was finding it difficult to work out what she needed and how to go about accessing those things. Seeing what was available to assist her recovery from the TAC encouraged her to reflect on her situation and better engage with the insurer to support her recovery:

“One of the things [the program] was really helpful with making me see was to do with saying, I’m not okay. I need help with… housework or gardening and things like that… It’s fairly normal part of a recovery process to get some assistance… Your mind doesn’t tend to really think of that when you are in the midst of it… You were supported to see different ways of looking after yourself.”

Simon discussed how the interactions with his clinician facilitator helped to understand the role his treating occupational therapist could play in his recovery, prompting him to get more out of the treatment.

“Now I know what the OT is supposed to be doing… I can start focusing on getting a little bit of my life back. That’s the biggest thing that I got out of it…[The facilitator] has given me the confidence…that it’s my right, I should be pushing for these things… In that way it’s given me more control”.

Despite the information being available on the website and some participants describing receiving information from the TAC in the mail shortly after lodging their claim, several participants described not being aware of the TAC’s app (myTAC) prior to watching the videos: *“It was really useful. When we went through the app again. No one in the TAC showed me that or how I could get an app on my phone… I didn’t even know I could do that.”* (Charles).

The clinicians who delivered the intervention felt that the combination of the information and the coaching approach to support the information was instrumental in improving participant self-efficacy:

“I like the content. If this would [be] available, I would like all TAC patients to be offered it as I feel it would greatly assist in reducing the potential for a descent into chronicity and may improve self-efficacy early in a patient’s injury experience.” (Clinician 1)

“[Participants] also gain an understanding of their rights in the TAC system and build skills in advocacy and self-efficacy. Both of these skills are required to manage the complex TAC system and manage pain.” (Clinician 3)

The information in the second video provided many strategies that a person could undertake to help them deal with their pain. While many participants reported that they already knew of the strategies, they would also often report that they had not actually used them or had forgotten to utilize them. Kate reported that the coaching prompts provided by the facilitator reminded her to incorporate these strategies into her day, which she found helpful:

“[I developed] better coping mechanisms. There were a couple of things I totally didn’t even think to try and [the facilitator] was really good at…[prompting] have you tried this? Have you tried that?”

For some, the pain education messages embedded in the videos, combined with reassurance from the facilitator helped provide reassurance and structure to their pain experience and recovery. Charles explained that despite being a healthcare professional himself and having some baseline knowledge of pain, the overwhelm he felt in his recovery period meant that he forgot some of the concepts and the reminders and reassurance helped reinforce this knowledge:

“It was a lot of talking about my pain and helping me understand what pain is. Which, again, I had preconceived ideas. I thought I would be fine and know it all, but I actually learned lots while I was doing it… I said to [the facilitator] I had your voice in my head telling me that, you know, this pain is okay.”

Other participants found that the facilitator encouraged them to use the pain management strategies presented in the video, such as pacing, to achieve tasks that were difficult for them. Alistair described how his facilitator encouraged him to get back in the garden, something he was wanting to do, but was finding difficult:

“[The facilitator said] get [your partner] to empty the mower but you push it around, so you are actually mowing the grass and you’re doing something that you enjoy. She said… start small… break it up… at the end of the day you are going to see that it looks nice and that will help you move.”

He went on to further describe how the pain management strategies had helped him to reduce his use of medication:

“[Without the program] I wouldn’t have done any of that. So I would have been sitting there just popping pain pills going… okay, I’m high as a kite but I’m not really doing anything to move forward.”

For Charles, the pain education messages combined with the coaching support helped build a sense of self-efficacy which helped to reduce distress.

“[I felt like] this is never going to get better… this pain is awful, and I can’t do anything. Then after having the sessions, I’m like, no, because I had this voice, I call in [the facilitator’s name] voice telling me that this is okay, that I just… need to do a little bit at a time.”

The sense of learning from the informational videos to be proactive in managing claims-related tasks, organizing treatment, and undertaking pain self-management approaches was demonstrated by nearly all the participants. In managing these elements on their own, claimants were seen to build a sense of control and confidence in their own ability to cope with the demands of the claim and the presence of pain. In many instances, developing higher self-efficacy appeared to lead to lower levels of distress.

#### Healing with Social Connection

Having a person who provided an empathic and non-judgemental listening ear and also understood the issues faced by people recovering from an injury and interacting with an insurer was reported to be of great benefit to most participants. It was also reported that the use of knowledgeable health professionals to deliver the intervention meant that participants felt reassured the information they received was trustworthy. Emma described the interactions with her facilitator to provide much comfort to her:

“[The facilitator] was always very supportive and very understanding of… what I’ve been through and what it’s caused… It felt like a… supportive friend… to talk about things. She had a good understanding of what I was dealing with.”

Alex also found the social support to be of benefit, particularly as it provided someone who was not a friend or family member to talk to:

“It helped me to keep a good perspective in terms of celebrating my achievements and milestones… I definitely found it helpful to have a completely separate person that I didn’t have to worry about, like, oversharing or burdening… It was some external way to help me process what I was going through.”

In describing how much she enjoyed the sessions, Andrea touched on the idea that recovering from an injury might also be occurring in the setting of dealing with the trauma of the accident and that support at this time was of great assistance:

“I’m a person living on their own, isolated… It was just someone to be checking that I was seeing the doctor, was doing this and knew this or that. [The facilitator] would really be that person that I [needed] with this amount of grief… I needed something like this [program]… I needed people who were experts.”

Charles also found the recovery period to be lonely and isolating and the contact with the facilitator was supportive:

“I genuinely found it super useful… because I was very isolated by myself. My husband had to go back to work financially. I was in the house by myself with two [broken] arms. It was actually nice to talk to someone who understood.”

With the facilitators being experienced clinicians, participants expressed that they were able to get some reassurance around elements of their recovery and that they valued the expertise and experience of their facilitator. Grace described:

“It was a lot of reassurance, like, it will get better… And I guess reassurance from [a person] in the medical field… he was pretty insightful for how it all kind of works.”

Alistair described how working with his facilitator made him feel more confident to continue along the recovery process:

“Talking to [the facilitator] helped me to contextualise a lot of it. Validate that I already had all the skills an individual can have for this situation.”

He went on to describe how the social connection had a positive influence on his mood:

“It definitely helped my mood. It definitely helped my headspace a little bit more. I was very excited [to have a session with the facilitator one afternoon]. Great! I can… explain how I’ve been feeling and what’s been going on… and know that at the end of that session she would provide me with some guidance – hey! Try these things, challenge yourself with these things.”

The feeling of having support where none had been forthcoming previously was powerful and, in one instance, life changing. When asked what her recovery might have looked like if she hadn’t had the intervention, Andrea replied:

“I don’t know. I was in such a state. I don’t know if I’d be here [without the program](crying)… I’m in such a different state… I think just getting that level of care after feeling that no care was coming from the system, you know.”

In a period of recovery where people were feeling vulnerable and potentially isolated, having an experienced clinician to provide social support was the most consistently described helpful aspect of the intervention for participants. The support was perceived by the participants as helping to improve mood, including reducing distress.

#### Further Along Than I Would Have Been

When reflecting on what their recovery might have looked like if they hadn’t participated in the intervention, many of the participants felt they would not have been as advanced in their recovery. This suggests that the intervention was instrumental in facilitating their recovery. Alistair reflected that without the intervention:

“I probably wouldn’t have been in the state that I am now.…Finding each of those things that were wrong and… doing something to help fix it.”

Both Kate and Grace suggested that while they know they would have been able to recover without the intervention, having the support meant that they were further along in their recovery than they otherwise would have been:

“[Without the program] I reckon [recovery] would have been a lot slower… I definitely think there would have been some disjointed things I just wasn’t aware of and instead of being pro-active, I would have been constantly trying to catch up on… what I should have done… I think it helped me fill in some of those gaps.” (Kate)

“I would have figured it out on my own, but it would have been a lot more of a frustrating process and it probably would have taken longer.” (Grace)

In reflecting on what recovery might have looked like without the program, many participants felt that they would have had further benefit if they had engaged with the program at an earlier phase in their recovery. They felt that, had they been able to access the information provided in the videos as well as the social support from the interactions with the facilitators, they may have been able to act on the information earlier to help with claims navigation or pain management strategies *(“I would have easily been in a better situation now if I found this program a month earlier,”* Alistair). Charles said of a discussion with his facilitator: *“The thing I said to her is, I wish you were here sooner, because maybe I would have treated my pain [better].”* In describing some of the shortfalls of her claims and recovery experience, Elizabeth said: *“Basically, I think if I got in contact with [the program] much earlier after my accident, it would’ve been maybe more beneficial to me.”* Similarly, when asked if there were possible suggestions for improvement to the intervention, Alistair replied: *“[It would be good] if this was available sooner and offered sooner.”*

Feeling satisfied that the program had been helpful in improving their outcomes, most participants said that they would recommend the program to others based on their experience. Simon described: *“Yeah, there’s not a price I can put on it. It’s more around the pride it gave me to get back to something. To own that part of my life again. So, yeah, definitely, I would [recommend it] hands down.”* Other participants were grateful to be included in the intervention. For example, Bob expressed: *“It just brought a whole lot of information to me. I really appreciate that. I appreciate your [intervention]. I appreciate your time.”* In thinking of who else the intervention might be helpful for, Grace suggested: *“It’s pretty lucky that I’m confident, I speak English, I’m not [elderly], I’m not vulnerable… This program would be really helpful for people who are… disadvantaged.”*

Participants in the study described being further along in their rehabilitation journey than they otherwise would have been, largely due to the pro-active approach they took to managing claims administration and their pain. In describing this, they also articulated a sense of satisfaction with the program and virtually all participants said that they would recommend the program to others who had experienced road traffic injury, confirming that the program was acceptable to them.

#### Telehealth Delivery was Acceptable and Feasible

The use of telehealth to deliver the intervention was reported by both claimants and clinicians to have contributed to the ease of engagement with the intervention. They suggested that being on telehealth removed potential barriers to engagement such as transportation availability and travel distance. For example, Charles said: *“It was all telehealth. But that was fine for me and actually worked better. Because of my arms, I couldn’t leave the house.”* Similarly, Alex reported: “*In terms of accessibility, it’s good to have it *via* Zoom. At the moment… to leave the house I have to have crutches.”* There was an overall sense that Zoom was easy enough to operate and that people were familiar with it. (Simon: *“A lot of my appointments… are telehealth now… I’m used to it.”*). Echoing the claimant experience, clinicians reported that the use of video conferencing was convenient and enhanced accessibility:

“The use of tele-health for this program was ideal, especially the flexibility it provides around delivery times and access to patients. [It allowed for] the ease of delivery of the content of the program.” (Clinician 1)

The choice to use telehealth with a live video component was described by participants as being preferable as it helped to create a connection with the facilitator. Grace described: “*I like Zoom more than the phone but that’s just because I’m used to Zoom… I like to have a face to talk to.”* Kate also felt the video brought connection to the consultations and described: “*At least you are having a face-to-face conversation which probably makes it a bit more personal. You probably… relate to other people and feel more comfortable… because you have got that little bit of interconnectedness which is a good thing.”*

There were few technical issues associated with the use of telehealth and any issues that did arise were able to be dealt with at the scheduled time of the consultation and it was subsequently able to be delivered. Overall, both claimant and clinician participants reported that the use of telehealth was both acceptable and feasible.

## Discussion

The present study demonstrated that a co-designed intervention to mitigate distress and support pain self-management early following road traffic injury was feasible to deliver and was acceptable to claimant participants. The intervention provided targeted information that enhanced participant’s knowledge and self-efficacy, and tailored social support for people who were at risk of a poor outcome following motor vehicle accident-related injury in Victoria, Australia. The use of a coaching approach by the clinicians seemed to be successful in encouraging claimants to take a more active role in their recovery and claims management and boosting self-efficacy, a known predictor of outcomes after compensable injury [9, 10, 57, 58]. Together with informational videos that supported claimants to better manage their claim and their pain, the intervention provided social support and information that was appreciated by participants in a time of vulnerability. The participants overwhelmingly expressed that they had found benefit in the intervention and that they would recommend the intervention to others who had experienced road trauma.

Delivery of the intervention was found to be acceptable and feasible. Clinicians reported that the use of telehealth was advantageous in providing flexibility and accessibility for delivery. Likewise, claimants enjoyed the telehealth design as it overcame barriers to accessing in-person care/support. There were few technical barriers to its use and clinicians reported high levels of engagement with the intervention by claimant participants. Feedback from claimant participants showed that they too appreciated the flexibility and accessibility afforded by the use of telehealth and they valued having a qualified allied health clinician as a consistent source of support.

The desire for social support in the early period following a motor vehicle accident was strongly expressed by former claimants during the co-design process. Claimant participants in this intervention overwhelmingly expressed that the presence of a consistent and trustworthy person who provided a listening ear and valuable encouragement was helpful and appreciated. Previous research has identified that perceived social support following traumatic injury is a strong moderator of outcomes. People who report low perceived social support associated tend to have higher levels of pain six weeks after injury [59] and worse functional and mental health outcomes following discharge [60, 61]. Additionally, in people recovering from transport-related musculoskeletal injury, the presence of higher levels of social support has previously been found to reduce healthcare service use [62]. The positive feedback from participants regarding the provision of social support in the intervention suggests that it helped meet the information needs for participants. Additionally, it may have played a role in the reported benefits including enhancing recovery. This study provides the necessary support and pilot data for future randomized controlled trials that can test the effectiveness of the intervention on outcomes such as healthcare utilization and function.

The use of standardized videos to deliver the information about claims management and pain self-management strategies is an example of the use of digital health technologies in healthcare. Digital health solutions such as informational videos, provide opportunities to deliver information that can be accessed outside of clinical consultations and can be shared with family and other caregivers. It offers convenience, standardization of information, and low-cost delivery [63–65].

Feedback obtained during the co-design process suggested that the sense of overwhelm and the presence of pain were both barriers to claimants engaging with informational resources, such as the TAC website on their own. It should be noted that the content of the videos was largely available to claimants on the TAC website as well as in an information pack provided to claimants on initiation of a claim. Additionally, there were potential opportunities for claimants to be exposed to information about the claims process and pain self-management strategies from interactions with TAC claims staff or with their clinicians. Despite all claimant participants having an accepted TAC claim and being under the care of a GP, and most were also seeing allied health providers, claimants reported that the information provided in the videos was in many instances new to them. It appears that the provision of targeted information in the form of the videos may go some way to fill the knowledge gaps expressed by participants in the co-design process. However, the feedback from both claimant and clinician participants of this study suggest that it was the combination of information as well as the encouragement from the clinicians that was pivotal in bringing about behavior change. This is supported by a systematic review that suggested that tailored information was of greater benefit than more generic educational information provided to people who have experienced road traffic injury [66]. In the present study, the combination of information and coaching led to claimant participants taking steps to action both claims-related tasks and engaging with pain-self management strategies, boosting self-efficacy. Future research should investigate the impact of providing informational videos alone versus the combination of videos with the support of a clinician coach to examine the effects on confidence to manage claims administration and pain.

Motivational interviewing methods [50, 51] were employed by clinicians to encourage claimant participants to take action to address issues that had been identified during their sessions. The clinicians were trained to encourage claimants to reflect on information provided in the videos or to come up with new strategies to resolve issues they were facing. This approach was designed to encourage participants to problem solve their own issues and has been successfully used in other early interventions [67, 68]. It was seen to encourage claimants to engage in tasks that they had not known to be necessary prior to watching the videos, had been unsure how to undertake, or had been putting off due to low motivation or uncertainty. As a result, participants described having greater confidence to manage their claim, suggesting that they were taking a more active role in their recovery journey. Further, in addressing certain administrative tasks in a timely fashion, claimants reported that they avoided potential delays to care. Delayed care in compensable settings has been described as a source of distress in previous research [2, 11, 28, 29, 69]. Higher levels of distress are known predictors of poor outcomes [11, 70–72]. Future research should investigate the long-term effects of improving self-efficacy in navigating an insurance claim on outcomes such as distress, healthcare utilization, and return to work.

### Implications

The present study highlighted the feasibility of delivering this tailored intervention to claimants of the TAC in Victoria. The helpfulness of the intervention described by participants was characterized by some elements that would be universal in any post-injury setting, namely consistent social support and pain-self management education. However, much of the feedback pertaining to the claims information provided in the intervention was specific to processes and requirements of the TAC claims process. As such, in order to adapt and scale this intervention to other settings, key stakeholders within that setting would need to be consulted and involved in the co-design. This would ensure issues experienced by claimants in specific insurance settings can be directly addressed.

The costs of delivering the intervention were limited to clinician costs (cost of care), video production costs (one-off cost), and training costs (one-off cost). There is some evidence to suggest that, in addition to improved functional outcomes [13–15, 73], early interventions may reduce total claims cost by reducing wage replacement and medical costs [13, 15]. Several participants in this study felt that without the program they would have experienced delays to care. Studies have reported that delays to care are considered to be a source of distress for claimants [11, 74] and hence if the intervention helps to prevent unnecessary distress, it would be a significant benefit for claimants.

### Strengths and Limitations

The use of allied health clinicians with expertise in pain management who had prior experience treating TAC claimants meant that clinicians had insight into the experiences of TAC claimants beyond what was provided in the informational video. They were able to use this prior experience and understanding when guiding claimant participants in addressing the issues they faced. The use of clinicians (as opposed to non-clinicians) also meant that participants felt comfortable discussing their injury recovery with their facilitators knowing that the advice and reassurance they received was trustworthy. Further, the clinicians’ professional experience and further education meant that they had the knowledge and skills to deal with high levels of distress that were occasionally experienced by claimant participants. While this was a strength for the present study, the availability of such highly trained clinicians may limit the scalability of such an intervention.

One author (AS) delivered the intervention to two participants. The potential limitation with this is that the author was also involved in the coding and thematic analysis of the data and as such, this experience may have biased her views on the program and the claimant experience.

The aim of the study was to determine the feasibility of the intervention and, as such, impacts on health outcomes were not examined using patient-reported outcome measures. The intervention was acceptable and feasible, and a larger scale trial is warranted to further evaluate the intervention. Our recruitment strategy also meant that participants who could not speak or understand English, or who had lower literacy or technical resources or skills (use of QR code or email) were less likely to be recruited to the study. Further, only one of the participants in the study was from a regional or rural area therefore it is possible that we have not captured the views of this sub-population adequately. As this study predominantly included English speakers living in metropolitan areas, the transferability of these findings to other settings or populations is unknown.

## Conclusion

The delivery of a co-designed telehealth-delivered intervention to support people with a compensable injury in a single insurance scheme was feasible and acceptable to people who were at risk of a poor outcome. Further research to evaluate the efficacy of the intervention on outcomes such as pain, self-efficacy, and claims costs is needed.

## Supplementary Information

Below is the link to the electronic supplementary material.

Supplementary file1 (DOCX 18 KB)

Supplementary file2 (DOCX 18 KB)

Supplementary file3 (PDF 92 KB)

Supplementary file4 (DOCX 14 KB)

Supplementary file5 (PDF 92 KB)

Supplementary file6 (PDF 52 KB)

## Data Availability

The datasets generated during and/or analyzed during the current study are available from the corresponding author on reasonable request.
