# Supplementary material for: Evaluation of the Acceptability and Feasibility of Stress Mitigation Education and Support Delivered via Telehealth for People After Road Traffic Musculoskeletal/Orthopedic Injury
Source: J Occup Rehabil. 2024 Nov 29;36(1):207–22. doi: 10.1007/s10926-024-10258-z (PMC12906523; doi:10.1007/s10926-024-10258-z)
Supplement: Supplementary file 1 — Supplementary file1 (DOCX 18 KB) [file 10926_2024_10258_MOESM1_ESM.docx]

Appendix A

Script for Informational video on TAC claims process

The Transport accident commission or TAC is Victoria’s government-owned, compulsory third party insurer. The TAC is a no-fault scheme, which means regardless of who is at fault in the accident, the TAC are there to help you get life back on track

TAC funding comes from the payments made by drivers when they register their vehicles each year through Vic Roads.

The TAC website has all the information that you need to know to manage your claim.

To make the process of managing your claim a bit easier, the TAC has an app called myTAC

If you find it hard to use apps or just prefer navigating the web on your computer, you can access the same information and services through the website (picture of webportal on website)

You can download the app via your app store and there are links on the website, (pictures of android or iphone shops)

It allows you to:

- - Send and receive messages about your claim
  - Submit receipts and claim reimbursements
  - Send documents to the TAC
  - Find information about what treatments and services the TAC may pay for

When you have an accepted TAC claim, in the first 90 days after your accident you don’t need TAC approval to access most tests or treatment that you need in order to recover from the injuries that you sustained in your accident. This might include physiotherapy, medical services, X-rays and scans or surgery if that is what is needed because of your accident.

There is a comprehensive list on the website (picture of the website with this section)

The cost of surgery and diagnostic tests related to your accident injuries are fully covered by the TAC.

When making appointments for other healthcare, for example, physiotherapy, osteopathy, chiropractic or exercise physiology, keep in mind that the TAC pays a standard rate for appointments. Some healthcare providers charge more than this rate. For those providers you may need to pay a gap between what the provider charges and what the TAC pays. If paying a gap is going to be difficult you may need to find someone who bulk bills. We suggest you search on the internet.

The good news is, most people will have their life back on track well within 90 days of their accident. In rare cases, it might take longer than this.

After 90 days, TAC can continue to pay for support from your GP and other providers, but you will need to seek approval from the TAC for this ongoing care.

If it looks like you are not going to be back on track within 90 days and you might need ongoing care from healthcare professionals beyond 90 days, your health care provider can support you in your application for further treatment. This approval process often takes between 2 and 4 weeks. In the rare case that you need ongoing treatment after 90 days, it can be helpful to get the process happening early so there is no interruption to your rehabilitation.

In general, the TAC are happy to approve evidence based treatments that are likely to help you recover.

The TAC understands that sometimes you might need a little bit of extra support. Talk to your client manager about what you need and they can tell you what is possible and the support the TAC can offer.

There is also more information about support options on the website.

The TAC can provide income support if you are unable to work for more that 5 days because of your accident-related injuries. This provides a safety net to help you recover.

Information about income support can be found on the website or with assistance from your claims manager.

At times, generally later in the claim, the TAC may ask you to attend an independent medical examination. These are not TAC doctors, but are specialist, independent doctors who provide TAC with a better understanding of your condition. This process is designed to ensure that you are being offered the best and most appropriate care.

If you ever need to contact the TAC by phone, keep in mind that wait times can be variable. We suggest calling first thing in the morning. Another good tip can be to put your phone on speaker or your headphones in and get other tasks done while you wait for your call to be answered.

= Another alternative to calling the TAC is to use the myTAC app, which has options for directly contacting the TAC. You can send a message directly to the person you wish to speak to, or you can request to be called back.

There are links to further support services, including the Amber community, a free telephone or face to face counselling service for people who have experienced trauma in a road accident.

There is lot a lot more information on the website to help you get life back on track.
