# Supplementary material for: Evaluation of the Acceptability and Feasibility of Stress Mitigation Education and Support Delivered via Telehealth for People After Road Traffic Musculoskeletal/Orthopedic Injury
Source: J Occup Rehabil. 2024 Nov 29;36(1):207–22. doi: 10.1007/s10926-024-10258-z (PMC12906523; doi:10.1007/s10926-024-10258-z)
Supplement: Supplementary file 2 — Supplementary file2 (DOCX 18 KB) [file 10926_2024_10258_MOESM2_ESM.docx]

**Appendix B**

**Script -Pain management information, Session 2**

It is normal to feel quite tender or sore when you have injuries following a motor vehicle accident. When we injure ourselves, our body produces an inflammatory response, which you might notice as swelling, redness or bruising. This is a healing response. It is designed to protect the area that is injured and promote healing. The inflammatory response is designed to be helpful but it can make the injured areas quite sensitive or painful.

There are many ways that we can manage pain while we recover.

Medications can help but we want to use them only as prescribed and only for as long as necessary. There is a lot that we can do apart from taking medications to reduce pain and the impact of pain on our lives. In this video we will share some strategies that you can use, anytime, anywhere, and for as long as you need, to help you manage your pain while you recover from injury

Pain can be reduced dramatically using distraction techniques. When you focus your attention away from your pain, it will be less bothersome. Reducing how much the pain bothers you, as much as reducing the amount of pain itself, changes how you “feel” or “experience” this pain and improves your ability to return to your normal activities more quickly. Here are some distraction techniques you can try when pain is intrusive.

 • Try counting forward or backwards by three (for example, 3-6-9-12-15-18, or 100-97-94-91).
 • Pick a letter and a topic, for example “L” and “food”. Bring to mind every food you can think of that starts with the letter “L”.
 • Watching the television can also help, particularly if you are interested in the show.

Listening to your favourite music can help lift your mood and distract you at the same time,

These distraction strategies reduce our experience of pain in two ways: First, they help us not to focus on unpleasant sensations. When we don’t pay attention to what is going on in our body, we are less likely to be bothered by it. Have you ever noticed, for example, that you don’t feel hungry when you are really interested in or busy with what you are doing? Your stomach might be empty, but we don’t feel the sensation of emptiness until we take a break from what we are focusing on.

The second way that distraction helps is by calming us down. Focusing your attention on a task that is either pleasant or just engaging helps to reduce feelings of distress. During your recovery period, you might find it helpful to distract yourself by:

 • Reading a book, magazine, the news, or short entertaining stories. • Listening to podcasts, or downloading your favourite book to your phone or other mobile device 
• Doing puzzles like crosswords or sudoku, or other games that can be downloaded to your phone or device.

- Doing hobbies such as knitting

The use of hot and cold packs can provide both distraction and pain relief. Discuss this with your healthcare providers to see if this is appropriate for your injuries and how you might best achieve this.

Having a chat with a friend or family member – even if by text or phone – is a great way to distract you from the feeling of pain or other discomfort. Partly, this is because texting or talking with a friend over the phone takes your mind off the pain. And in part, it is because talking with a friend or family member makes us feel safe and supported. Connecting with others who make us feel valued and safe also feels good – we get a shot of natural pain killers called endorphins when we interact with others – even if they are strangers.

Endorphins are the body’s natural pain killers and we can increase our body’s production of endorphins in many ways. Sitting or walking in the sunshine, talking with a friend, acquaintance, or thinking about what you will do when you have healed can all help with the release of endorphins. Knowing that you are helping your recovery is also important in turning on your ‘inner pharmacy’. You can also increase your body’s endorphins by doing light exercise within the limits of your injury and as recommended by your health professionals, giving or receiving physical comfort from a friend, partner, pet, or family member, and getting fresh air out in nature.

Our tolerance for pain and our pain sensitivity changes from day to day. It can be influenced by your mood or emotions like stress or anxiety, regardless of the cause of the pain. Many people find that if their mood is good, they feel more positive about their recovery and they are better able to tolerate pain. Your mood might be affected by what you read or listen to, who you talk to, what you think about, or even what you eat. What affects your mood? Be mindful of what affects your mood, and try to do something each day that makes you feel good. It will help you to manage pain and help you to recover faster.

It is normal to feel worried or anxious after an injury. However, stress causes tension, and can increase your pain, so it is important to deal with stress and worry. It can be helpful to talk to someone about your feelings – a friend, family member, or if you are concerned about something to do with your injury or your recovery, then speak with your healthcare providers.

If the idea of talking about your feelings doesn’t sit well with you, the good news is that you can actually get a lot of benefit just from sitting in the company of others. Just being in the presence of people you trust or people who make you feel good can help with pain. This is because when we are in the company of people we like, we start to feel relaxed. Relaxation is a great strategy for managing pain.

You can also help yourself to relax by breathing out as slowly as you can after a short inhale. Slow, deep, exhales will relax your body, and reduce tension in your body. Another helpful strategy for relaxing your body is progressive muscle relaxation. You can best do this while you are lying down, and it may be useful in helping you to get off to sleep at night. Progressive muscle relaxation involves focusing your attention on one muscle at a time, tensing that muscle intentionally, holding that tension for a moment, and then intentionally relaxing the muscle. Often, we don’t even realise how tense we are until we focus our attention on a particular part of our body and make a conscious decision to relax those muscles. The process of progressively tensing and releasing the muscles of the body, from head to toe, places the body in a deep state of relaxation. It can be useful to focus on areas other than your injuries to help relax and settle. 

You might also find that the way that you think about pain influences how much it bothers you. It is important to remember that pain is not always a sign of harm. In fact, pain is an important component of recovery. It protects us from harm by keeping us from overdoing things. Rest is important, but don’t forget to keep moving in ways that are appropriate for you and as suggested by your healthcare providers. Movement is a really important part of the healing process and will help with your recovery.

In summary, we manage pain after injuries in different ways. You may be provided you with medications, but pain management is most effective when you combine medications with other techniques such as distraction, social connection, mood management, relaxation, seeking social support and connection, doing things to increase your body’s natural endorphins, and changing the way you think about pain. Rest assured, while injuries may take some time to heal, if you use these techniques, your pain will improve much faster.
