# Supplementary material for: Evaluation of the Acceptability and Feasibility of Stress Mitigation Education and Support Delivered via Telehealth for People After Road Traffic Musculoskeletal/Orthopedic Injury
Source: J Occup Rehabil. 2024 Nov 29;36(1):207–22. doi: 10.1007/s10926-024-10258-z (PMC12906523; doi:10.1007/s10926-024-10258-z)
Supplement: Supplementary file 3 — Supplementary file3 (PDF 92 KB) [file 10926_2024_10258_MOESM3_ESM.pdf]

Welcome to the program. We will be in touch shortly to connect you with your facilitator, who will guide you through the program.

What is involved in the program?

You will meet with your facilitator once a week for five weeks over Zoom. The program is designed to provide additional support to help you through the early phases of your recovery, including managing your claim. Your facilitator is there to provide a listening ear and help you access the support that you need during this time. All of our facilitators are experienced allied health clinicians who have worked with many people who have experienced motor vehicle accidents.

Your healthcare

This program is designed to run alongside your existing healthcare treatment. It is not designed to replace treatment from your existing or local healthcare providers. Please ensure that you maintain contact with your GP throughout the length of your claim.

Friends and Family

The time following injury can be challenging, and some people might like a friend or family member to attend the sessions with them to help with remembering the information. You are welcome to invite someone to join you if you feel this will be helpful.

Using Zoom

If you haven't used Zoom before, you will need to download the app from your app store prior to your first session. Your facilitator will provide you with a link for your sessions prior to the first session. If you need help getting this set up, please email: asim6928@sydney.edu.au

Accessing resources

During the program, some videos will be shown to provide some information that may be of interest to you. We know that it can be hard to take in all the information at once, so you can access these videos at any time after they have been shown to you by clicking this link: Alison TAC Video with Subtitles.mp4

There is no need for you to watch the videos ahead of time. They will be shown to you during the sessions. Links to other resources that may be of help can also be found through the above link.

Cancellations

Please ensure that if you need to cancel your appointment you give your facilitator 48 hours notice. We understand that life happens and that things can be a bit uncertain in this time following injury however we would be grateful if you can be conscious of attending your

appointment on time and letting the facilitator know if you need to change your appointment time.

Final interview

Following the final session, we would like you to participate in a feedback interview so that we can get a good understanding of your experiences with the program. This feedback will help us to make changes and improvements to the program. This interview will also be over Zoom and will go for around 30 minutes. A researcher will be in contact following your final session to organise a time for this.

Contact

If you have any issues during the program and would like to speak to someone, you can email asim6928@uni.sydney.edu.au or claire.ashton-james@sydney.edu.au

It is important that if you have any issues relating to your health that you connect with your local healthcare providers.
