# Supplementary material for: Evaluation of the Acceptability and Feasibility of Stress Mitigation Education and Support Delivered via Telehealth for People After Road Traffic Musculoskeletal/Orthopedic Injury
Source: J Occup Rehabil. 2024 Nov 29;36(1):207–22. doi: 10.1007/s10926-024-10258-z (PMC12906523; doi:10.1007/s10926-024-10258-z)
Supplement: Supplementary file 4 — Supplementary file4 (DOCX 14 KB) [file 10926_2024_10258_MOESM4_ESM.docx]

**Appendix D**

**Interview guide**

**Introduction:**

Thank you for taking the time to participate in this part of our study. Your feedback on the program is valuable to us as it helps us to understand your experience. This interview is being recorded with audio only and will be transcribed.

**Verbal consent:**

Do you give your consent for this to be recorded, transcribed and used as part of the study?

**Questions:**

1. Tell me about your experiences with the program
2. Would you recommend this program to others? Please tell us why or why not
3. Did you have any difficulty attending the appointments? Please tell us about this
4. Do you have any suggestions for how the program could be improved?
5. Was the program helpful in any way? Please tell us about this
