# Supplementary material for: Evaluation of the Acceptability and Feasibility of Stress Mitigation Education and Support Delivered via Telehealth for People After Road Traffic Musculoskeletal/Orthopedic Injury
Source: J Occup Rehabil. 2024 Nov 29;36(1):207–22. doi: 10.1007/s10926-024-10258-z (PMC12906523; doi:10.1007/s10926-024-10258-z)
Supplement: Supplementary file 6 — Supplementary file6 (PDF 52 KB) [file 10926_2024_10258_MOESM6_ESM.pdf]

Feedback survey

Tell us about your experience with delivering the program

Would you recommend this program to patients? Please elaborate

Would you recommend this program to colleagues to deliver as a facilitator? Please elaborate

What was your experience with participants level of engagement with the program? Please elaborate on why engagement was high or low

Did you have any suggestions as to how the program could be

improved?

Powered by Qualtrics
